# Illustrations of Unconscious Memory in Disease, Including a Theory of Alteratives

**Published:** 1886-09

**Authors:** 


ILLUSTRATIONS OF UNCONSCIOUS MEMORY IN DISEASE, INCLUDING A THEORY OF ALTERATIVES. By Charles Creighton, M. D. New York: J. H. Vail & Co. 1886.

It is difficult to believe that the author of this book intended that it should be taken in sober earnest. It is one of the most typical examples that could be imagined of the *a priori* method of reasoning in which words, ideas and facts are all bent to correspond with and corroborate a preconceived theory; yet, so ingenious is the sophistry and so apt the citation of proofs, that the book is interesting from beginning to end, and he who takes it up will be loth to lay it down unfinished. It is like one of Jules Verne's novels, in which, in spite of the absurdities and impossibilities, one is fascinated by the ingenuity manifested in the manner of their presentation.

The author starts out by establishing an entirely new and artificial definition of memory, and supposes the existence of a function in man to correspond to this new definition. He then draws a parallel between this imaginary function and certain physiological and pathological phenomena, and labors to prove that such phenomena are dependent upon the existence of the unction in question. If he has not succeeded in proving his theory to the satisfaction of the average reader, he has at least produced a readable book, and one which will repay perusal. At the same time we cannot help thinking that so much study and ingenuity might have been more profitably expended in other directions. V. O. H.

